# Federated SPARQL query performance evaluation for exploring disease model mouse: combining gene expression, orthology, and disease knowledge graphs

**DOI:** 10.1186/s12911-025-03013-8

**Published:** 2025-05-16

**Authors:** Tatsuya Kushida, Tarcisio Mendes de Farias, Ana C. Sima, Christophe Dessimoz, Hirokazu Chiba, Frederic B. Bastian, Hiroshi Masuya

**Affiliations:** 1 BioResource Research Center, RIKEN, Tsukuba-shi, Japan; 2 SIB Swiss Institute of Bioinformatics, Lausanne, Switzerland; 3 University of Lausanne, Lausanne, Switzerland; 4 Database Center for Life Science, DS, ROIS, Kashiwa-shi, Japan

**Keywords:** Database integration, Gene-disease association, Gene expression, Knowledge graph, Model organism, Ontology, Orthology, RDF, SPARQL

## Abstract

**Background:**

The RIKEN BRC develops and maintains the RIKEN BioResource MetaDatabase to help users explore appropriate target bioresources for their experiments and prepare precise and high-quality data infrastructures. The Swiss Institute of Bioinformatics develops two databases across multi-species for the study of gene expression and orthology: Bgee and Orthologous MAtrix (OMA, an orthology database).

**Methods:**

This study combines the RIKEN BioResource data with Resource Description Framework (RDF) datasets from Bgee, a gene expression database, the OMA, the DisGeNET, a human gene-disease association, Mouse Genome Informatics (MGI), UniProt, and four disease ontologies in the RIKEN BioResource MetaDatabase. Our aim is to evaluate the distributed SPARQL query performance when exploring which model organisms are most appropriate for specific medical science research applications across the aforementioned interoperable datasets. More precisely in our biomedical use cases, we investigate disease-related genes, as well as anatomical parts where these genes are expressed and subsequently identify appropriate bioresource candidates available for specific disease research applications.

**Results:**

We illustrate the above through two use cases targeting either Alzheimer’s disease or melanoma. We identified 14 Alzheimer’s disease-related genes that were expressed in the prefrontal cortex (e.g., APP and APOE) and 55 RIKEN bioresources, which were genetically modified mice related to these genes, predicted to be relevant to Alzheimer’s disease research. Furthermore, executing a transitive search for the Uberon terms by using the Property Paths function, we identified 14 melanoma-related genes (e.g., HRAS and PTEN), and 12 anatomical parts in which these genes were expressed, such as the “skin of limb” as an example. Finally, we compared the performance of the federated SPARQL query via the remote Bgee SPARQL endpoint with the performance of a centralized SPARQL query using the Bgee dataset as part of the RIKEN BioResource MetaDatabase.

**Conclusions:**

As a result, we confirmed that the performance of the federated approach degraded. We concluded that we reduced the degradation of the query performance of the federated approach from the BioResource MetaDatabase to the SIB by refining the transferred data through a subquery and enhancing the server specifications thereby optimizing the triple store query evaluation.

**Supplementary information:**

The online version contains supplementary material available at 10.1186/s12911-025-03013-8.

## Introduction

Bioresources are biological materials used for experimental life science research. They are widely used to elucidate the mechanisms of biological processes, including functional analyses, drug discovery, breeding, and practical chemical compound production as examples. Researchers generally source their bioresources from dedicated centers worldwide. These bioresource centers must develop retrieval systems to help users explore appropriate target bioresources for their experiments and to prepare precise and high-quality data infrastructure.

The BioResource Research Center (BRC) at the Japanese Institute of Physical and Chemical Research (RIKEN) is one of the largest and most comprehensive resource centers and manages a wide array of bioresources, such as experimental mouse strains, cultured cell lines and genetic material of human and animal origin, plant seeds, and microorganisms. The mission of the BRC is to contribute to the improvement of living standards, and the development and prosperity of human beings through distribution of its bioresources. These bioresources are developed and prepared under rigid quality control, so as to provide reliable infrastructure to firmly underpin life science research development. For its bioresource data infrastructure, RIKEN BRC adopted the Resource Description Framework (RDF), due to its advantages for data interoperability and its current adoption by institutions of the BRC’s interest for reuse. RIKEN BRC is working to continuously provide high-quality information by developing metadata and knowledge graphs (KG) and providing information retrieval systems. In order to leverage bioresources, we have been effectively building interconnected KGs by integrating bioresource data with datasets of cutting-edge research results provided by external institutions. However, efficiently retrieving the relevant information from a KG presents technical challenges. These challenges include infrastructure development, building and maintaining a KG that encompasses database integration, and writing complex technical queries.

**Fig. 1:**
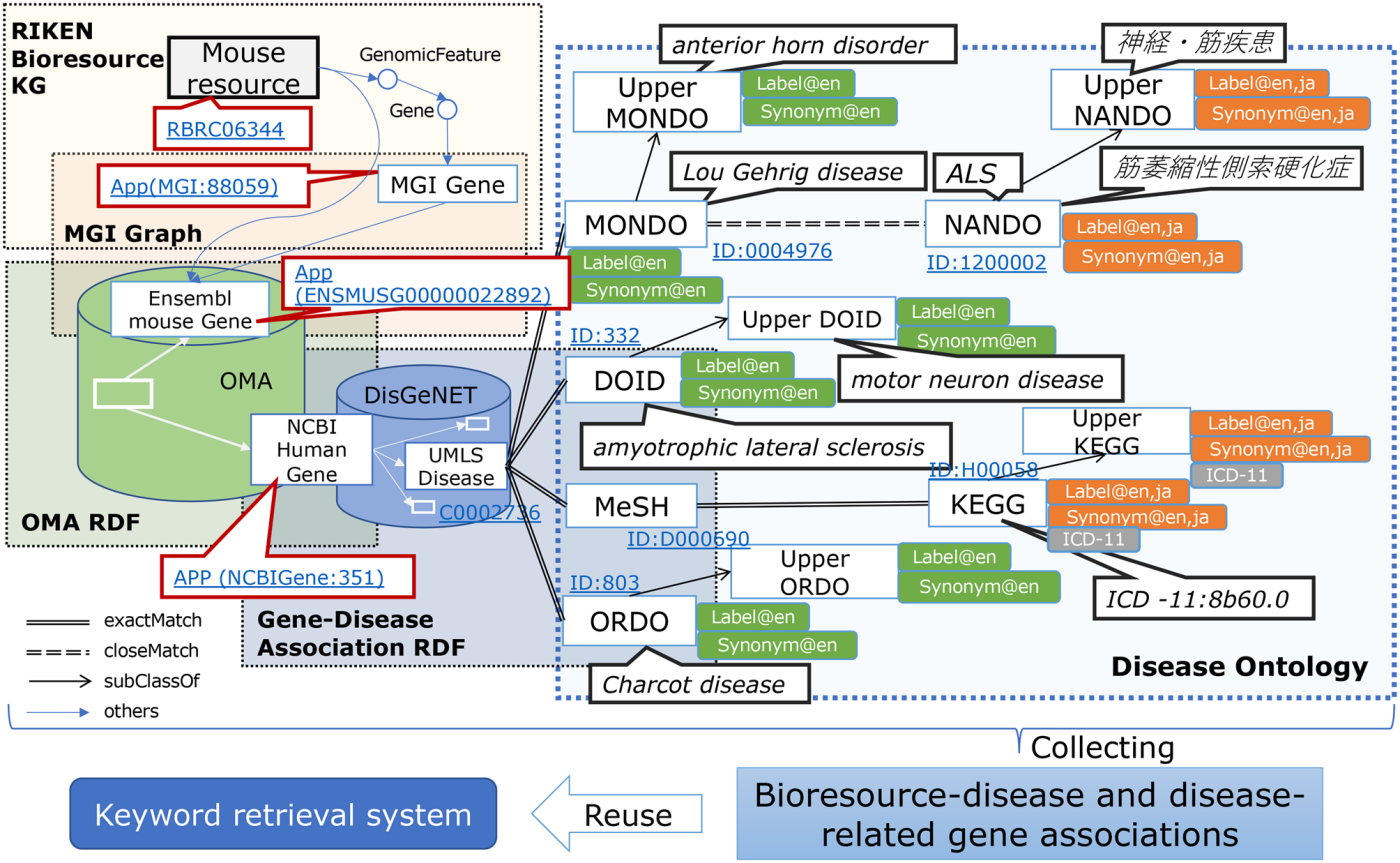
Data schema of RIKEN BioResource RDF data integrated with external RDF data and ontologies

The RIKEN BRC develops and maintains the RIKEN BioResource MetaDatabase (MetaDB) [1, 2]. This database integrates RIKEN BioResource RDF data with several life science datasets to support researchers in making a comprehensive use of RIKEN BRC’s research results. We call the integrated bioresource data the “RIKEN Bioresource Knowledge Graph.” So far, we have integrated the KG with the Orthologous MAtrix (OMA) database [3], DisGeNET [4], and disease ontologies, including MONDO Disease Ontology [5], Human Disease Ontology (DOID) [6], Orphanet Rare Disease Ontology (ORDO) [7], and Nanbyo Disease Ontology (NANDO) [8], which are provided by external organizations (Fig. 1). As a result, we are able to fully explore RIKEN BRC experimental mice, cell lines, and genetic materials available for research purposes [9].

The SIB—Swiss Institute of Bioinformatics develops and maintains a growing catalog of publicly accessible KG across many disciplines in the life sciences. For this study, we used two of the SIB RDF datasets for the study of gene expression and orthology: Bgee and OMA. Bgee [10] is a well-established gene expression database that integrates curated healthy wild-type expression data across a wide range of data sources to provide a comparable reference of normal gene expression across multiple animal species. OMA [3] (Orthologous MAtrix) is a database of orthologs among complete genomes across a wide range of species spanning the entire tree of life. Orthologs are pairs of genes that have evolved from a single gene in their last common ancestor. The OMA database provides orthologous information in the form of Hierarchical Orthologous Groups (HOGs), which are defined as gene families that contain genes that are all homologous to each other. The RDF version of OMA relies on the ORTH Ontology [11, 12].

In this article, we present a case study that explores candidate mice expected to be used for human disease research (disease model mice). To do so, we used a large KG consisting of bioresources, OMA for human-mouse orthologs, DisGeNET for associations between human genes and diseases (GDA), and gene expression data (Bgee). Specifically, we focus on creating federated SPARQL queries utilizing Bgee’s information on gene expression sites and expression levels of genes associated with human diseases, which are related to RIKEN’s genetically modified mice. Furthermore, we evaluated the search performance of these queries and examined the effectiveness of federated searches, which are expected to enable real-time searches against the latest data from the original data sources, and considerably reducing maintenance efforts on keeping data up–to-date. The rest of the paper is organized as follows: section “Related Work” reviews related work describing representative KG development cases and research in the life sciences. Section “RIKEN Bioresource Knowledge Graph” explains the RIKEN Bioresource KG, and section “Data Integration and Interoperability” presents external datasets and ontologies integrated with the RIKEN Bioresource KG. In section “Exploring Bioresources Relevant to Human Diseases” we performed SPARQL queries to retrieve bioresource candidates suited to a given disease research application. Section “Comparison Between Federated and Centralized Query Performance” presents the performance comparison results using the remote (federated) query over Bgee’s official SPARQL endpoint compared to using the local datasets (centralized) in BioResource MetaDB over a set of representative SPARQL query examples. Section “Discussion” discusses the outcomes acquired using the integrated KG, a revealed issue, and a solution. Section “Future Work” outlines future work.

## Related work

The Monarch Initiative is an international consortium working to expand the use of genome information in biology and biomedical research. The Monarch Initiative publishes RDF data related to bioresources [13]. The published RDF data include relationships between mouse genes provided by Mouse Genome Informatics (MGI) [14] and related diseases and genome variation data. However, the Monarch initiative does not provide an official SPARQL endpoint, and users need to implement the triple store themselves to use the RDF data.

Research on optimizing distributed SPARQL queries is essential for efficient data access and processing. DARQ [15] adopts heuristic–based approaches that generate query plans based on empirical rules and prior knowledge, such as the statistics provided in the service descriptions. It also employs dynamic programming and uses cost models to optimize query plans to some extent, similar to SPARQL-DQP [16]. FedX [17] uses heuristic–based approaches that do not rely on statistical data to generate query plans. SPLENDID [18] primarily uses statistics from VoID descriptions and cost models to optimize query plans. HiBISCuS [19], SemaGrow [20], and CostFed [21] primarily use cost models to optimize query plans, but there are subtle differences in their approaches. HiBISCuS is characterized by a novel source (e.g., datasets, and SPARQL endpoints) selection approach, while SemaGrow and CostFed focus on cost evaluation based on statistical information. Odyssey [22] employs dynamic programming instead of heuristics to break down the query into manageable subqueries, which are then solved optimally and combined to form the final result. FedUP [23] optimizes SPARQL queries by generating Result-Aware Query Plans based on query results, ensuring high performance even in large-scale SPARQL federations. Although, these approaches are applied for optimizing federated query plans, we do not apply them to our case study because of several reasons: the majority of them are not mature enough (i.e., either a proof-of-concept or a prototype) and mostly focus on data source selection instead of federated join operations; lack of support by the original SPARQL 1.1 endpoints (e.g., absence of VoID descriptions); there are no guarantees that they will improve the query plan of federated join operations of complex queries such as demonstrated in experiments in [23]. In [23], without any query federation optimizer, hand-crafted SPARQL 1.1 queries perform either better or slightly worse than the others for complex, multi-domain and cross-domain queries. Finally, in these experiments, all federation graphs are stored as named graphs in a single triple store endpoint in contrast to our use case where the graphs are stored in different endpoints across the globe. Therefore, in our work we provide a real-world practical case study that can contribute to development of a next generation of SPARQL federated query optimizers focusing on improving distributed join operations.

The Knowledge Graph Hub (KG-Hub) [24, 25] is a collection of biological and biomedical Knowledge Graphs, including their component data sources. It is provided by the Berkeley Bioinformatics Open-source Projects (BBOP) of the Lawrence Berkeley National Laboratory. KG-Hub tools comprise kghub-downloader, Koza (for data transformation), and KGX (Knowledge Graph Exchange), and KG-Hub uses these tools to transform data sources into standalone Biolink Model [26] compliant graphs. KG-Hub currently includes seven biomedical KG projects, including KG-COVID-19 [27] and KG-OBO [28]. The above-mentioned Monarch KG is also developed using these KG-Hub tools. KG-OBO translates the biological and biomedical ontologies on OBO Foundry [29] into graph nodes and edges. Ontology graphs translated by KG-OBO include Gene Ontology [30], ChEBI [31], and Uber-anatomy ontology (Uberon) [32].

Ubergraph [33] is an RDF triple store which provides a SPARQL query endpoint to an integrated suite of OBO ontologies, and includes precomputed inferred edges allowing logically complete queries over those ontologies for a subset of axioms in the Web Ontology Language (OWL) [34], and allows users to more efficiently access the integrated semantic knowledge graph. Ubergraph currently includes 39 OBO ontologies, including GO, ChEBI, Uberon, Cell Ontology (CL) [35], Mammalian Phenotype Ontology [36], and Human Phenotype Ontology [37].

## RIKEN Bioresource Knowledge Graph

**Fig. 2:**
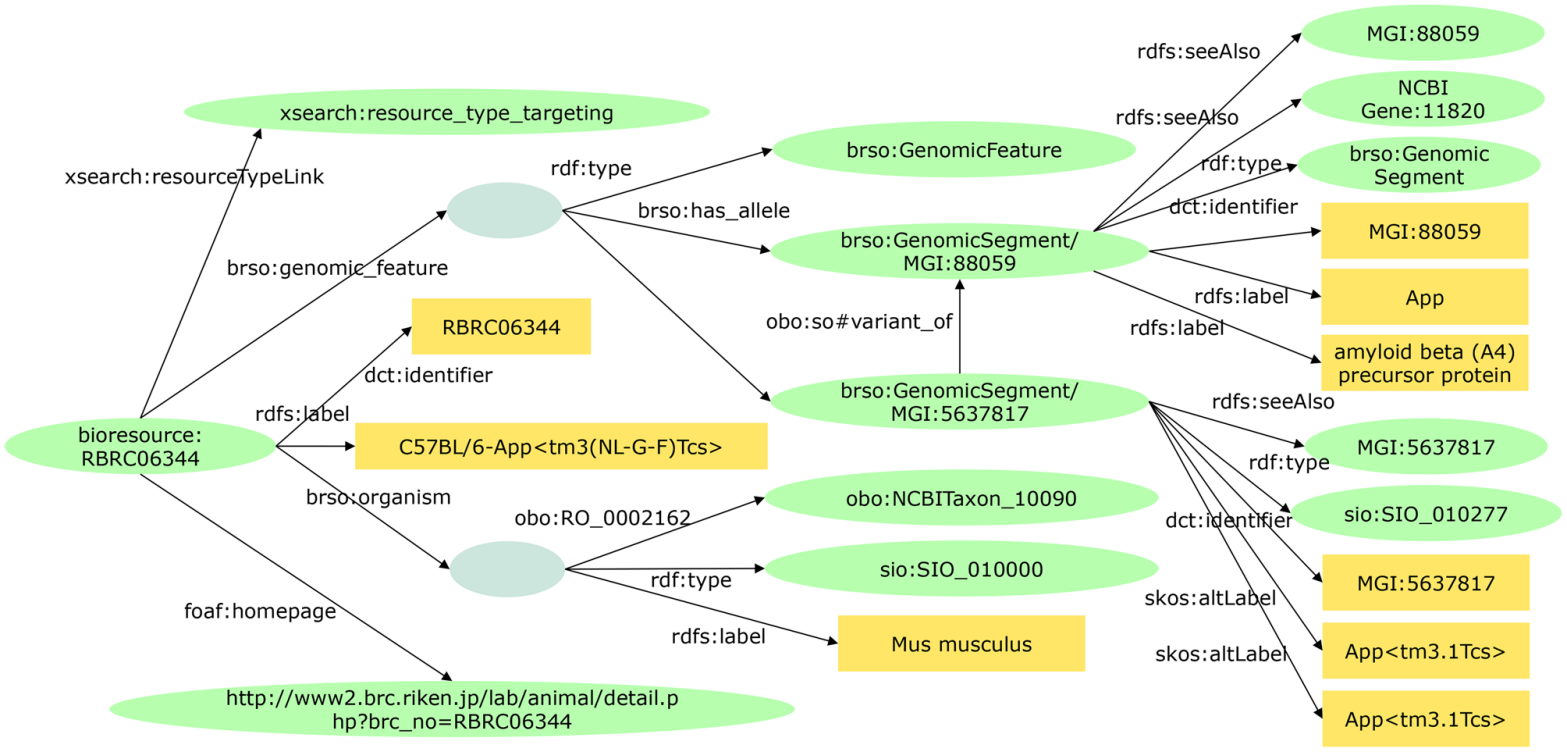
A part of RIKEN mouse (RBRC06344*) RDF data (KG) developed based on BRSO. *: https://knowledge.brc.riken.jp/resource/animal/card?__lang__=en%26brc_no=RBRC06344

RIKEN BRC publishes metadata related to managed experimental animals, cell lines, genetic materials, experimental plants, and microorganism strains on its webpage [38]. Furthermore, the BRC is also developing RDF data and integrating bioresource metadata with external public datasets to enhance information and knowledge relevant to these bioresources. The Biological Resource Schema Ontology (BRSO) [39] is an RDF data model for various model organisms and the types, such as individual, cell, and DNA, which is largely developed by the Database Center for Life Science (DBCLS), RIKEN, and National Institute of Genetics (NIG). RIKEN BRC is developing bioresource RDF data based on the BRSO (Fig. 2) [2].

We term the RIKEN BRC bioresource RDF datasets the “RIKEN Bioresource Knowledge Graph.” The KG contains administrative information (e.g., bioresource developers, their affiliation), organisms (e.g., Mus muscles), bioresource types (e.g., spontaneous mutation mouse), gene id (e.g., MGI:94859), the related phenotypes and diseases [e.g., amyotrophic lateral sclerosis (ALS)]. To date, we have developed KGs containing approximately 7800 experimental mice, 9600 cell lines, 125,000 genetic materials, 290,000 experimental plants, and 19,000 microorganisms. Users can browse KG data through a web interface, execute SPARQL queries, and download all the data from the BioResource MetaDB [40].

## Data integration and interoperability

We are integrating the RIKEN Bioresource KG with external public datasets to enhance information and knowledge relevant to bioresources. Because almost all users are experimental researchers, the data retrieval system needs to enable researchers to explore candidate bioresources through a search of the KG using their familiar identifiers or keywords, such as MGI, NCBI, Ensembl Gene IDs and UniProtKB accession numbers. We therefore enhanced the KG to integrate the following information and knowledge.

### MGI gene ID, Ensembl and NCBI gene ID mapping datasets

**Fig. 3:**
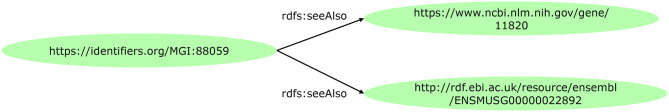
An example of RDF mapping data among MGI Gene, NCBI Gene and Ensembl Gene

**Fig. 4:**
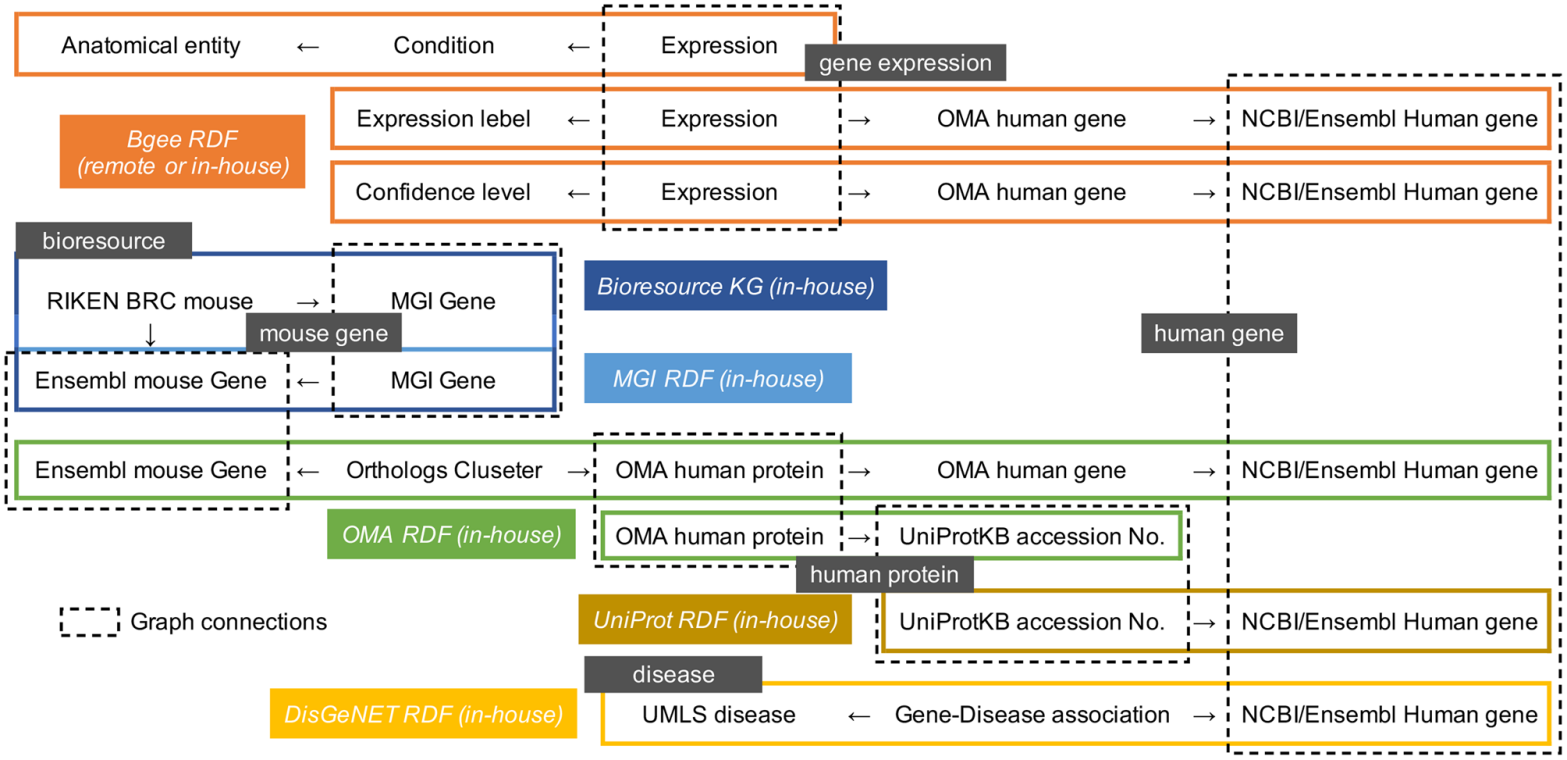
A simplified visualization of the query graph patterns

We developed RDF data representing relationships among MGI Gene ID, NCBI Gene ID, and Ensembl Gene ID from MGI Marker associations to Entrez Gene (tab-delimited) [41] provided from the MGI download page (Fig. 3). We stored the RDF data as a named graph in the BioResource MetaDB (Fig. 4). As a result, we could identify relationships between mouse resources, such as gene-modified mice and related NCBI Gene IDs and Ensembl Gene IDs in addition to MGI Gene IDs.

### UniProtKB accession number and NCBI gene ID mapping datasets

We developed RDF data representing relationships between the UniProtKB accession number and the NCBI Gene ID based on tab delimited files provided by UniProt [42]. We stored the RDF data as a named graph in the BioResource MetaDB (Fig. 4). As a result, we could identify relationships between mouse resources, such as gene-modified mice and related UniProtKB accession numbers, in addition to gene IDs.

### OMA RDF datasets

We integrated the Bioresource KG with ortholog RDF datasets: OMA developed and provided by the Swiss Institute of Bioinformatics (SIB) as a named graph (Figs. 1 and 4). This allowed us to acquire information on human Ensembl and NCBI gene IDs and UniProtKB accession numbers from gene-modified mouse gene IDs and UniProtKB accession numbers that are orthologous to human genes and proteins.

### Bgee RDF datasets

We integrated the Bioresource KG with the gene expression RDF dataset Bgee, developed and provided by SIB as a named graph (Fig. 4). As a result, we could access information on gene expression patterns, confidence levels and expressed anatomical parts from human Ensembl and NCBI gene IDs and UniProtKB accession numbers.

### Gene-disease association RDF datasets

We integrated the Bioresource KG with human gene-disease association RDF datasets: DisGeNET and MedGen as named graphs [43] (Figs. 1 and 4). The former was developed by the Institute for Research in Biomedicine (IRB, Barcelona), and the latter was developed by National Center for Biotechnology Information (NCBI), and the RDF data were generated and provided by DBCLS. This study used the GDA datasets of which the GDA score was 0.5 or more extracted from DisGeNET RDF v7.0.0 in the RDF Portal [44, 45]. As a result, we could access information on related human disease identifiers, such as UMLS IDs or MedGen IDs [e.g., C0002736, amyotrophic lateral sclerosis (ALS)] from human Ensembl and NCBI gene IDs and UniProtKB accession numbers.

### Disease Ontologies

We incorporated the OWL version of four disease ontologies that are used as controlled vocabularies: MONDO [5], DOID [6], ORDO [7], and NANDO [8] as named graphs, into the BioResource MetaDB (Fig. 1). The Monarch Initiative developed MONDO. The University of Maryland mainly developed DOID. ORDO was mainly developed by the National Institute of Health and Medical Research (INSERM) and the European Bioinformatics Institute (EBI). NANDO was mainly developed by DBCLS and RIKEN. As a result, we could access information on related human gene IDs from English and Japanese disease names, Disease Ontology IDs, and ICD-11 (International Classification of Diseases 11th Revision) [46] through these ontologies and DisGeNET.

## Exploring bioresources relevant to human diseases

In this study, we aim to identify disease-related genes, the anatomical parts where the genes were expressed, and the RIKEN bioresource relevant to the disease, by exploring the extended Bioresource KG using SPARQL queries. We applied this to two concrete use cases, targeting the study of Alzheimer’s disease and melanoma.

**Example 1-1**: Federated query for Alzheimer’s disease (see Additional file 1) is a query for exploring AD- (UMLS:C0002395) related genes expressed in specific anatomical parts (e.g., prefrontal cortex) and the bioresources expected to be available for AD research. This study partially revised SPARQL queries used in our previous report [47] to improve the query performance and executed the revised queries in the SPARQL endpoint [48] of the RIKEN BioResource MetaDB. The executed query included these query conditions: the prefrontal cortex (UBERON:0000451) as location of gene expression, a high confidence level for expression data, and the sex condition for “any sex type”. The strain type and developmental stage were not specified. We used the DisGeNET as gene-disease association datasets with the GDA score [4] of 0.5 or more.

We present the query results in Table 1. We identified that the 14 AD-related genes including APP gene (ENSG:00000142192) and APOE gene (ENSG:00000130203) and 55 RIKEN mouse resources expected to be of relevance for AD research including RBRC06344 and RBRC03390. APP and APOE genes have previously been linked to experimental AD, as reported in [49, 50]. The query runtime was over 600 s (Table 2).

**Table 1:** Results of Example 1-1: federated query for Alzheimer’s disease and Example 2-1: centralized query for Alzheimer’s disease

Query approach	No. of retrieved mice	No. of retrieved genes	Gene labels (ensembl gene IDs)
Example_1-1: Federated query for AD	55	14	PICALM (ENSG00000073921)
			PSEN1 (ENSG00000080815)
			NPY (ENSG00000122585)
			APOE (ENSG00000130203)
			APP (ENSG00000142192)
			PSEN2 (ENSG00000143801)
			ACE (ENSG00000159640)
			INSR (ENSG00000171105)
			BCL2 (ENSG00000171791)
			BDNF (ENSG00000176697)
			MAPT (ENSG00000186868)
			CD2AP (ENSG00000198087)
			INS (ENSG00000254647)
			Novel protein (ENSG00000288674)
Example_2-1: Centralized query for AD	55	14	PICALM (ENSG00000073921)
			PSEN1 (ENSG00000080815)
			NPY (ENSG00000122585)
			APOE (ENSG00000130203)
			APP (ENSG00000142192)
			PSEN2 (ENSG00000143801)
			ACE (ENSG00000159640)
			INSR (ENSG00000171105)
			BCL2 (ENSG00000171791)
			BDNF (ENSG00000176697)
			MAPT (ENSG00000186868)
			CD2AP (ENSG00000198087)
			INS (ENSG00000254647)
			Novel protein (ENSG00000288674)

**Table 2:** The query execution time of Examples 1-1, 2-1, 3-1, and 4-1. The queries were executed 10 times each at https://knowledge.brc.riken.jp/sparql

Query approach	Mean of runtime	No. of retrieved mice	No. of retrieved genes (disease related genes)
Example 1-1: Federated query for AD	> 600 s	55	14
Example 2-1: Centralized query for AD	**307 s**	55	14
Example 3-1: Federated query for melanoma	> 600 s	102	14
Example 4-1: Centralized query for melanoma	**502 s**	102	14

Values in bold indicate the shortest mean runtime for each disease context

In this study, we ran SPARQL query tests and obtained the retrieval results and runtimes on 4 August 2023 and 6 June 2024.

## Comparison between federated and centralized query performance

Furthermore, we evaluated two query execution scenarios [47]. One scenario considers a SERVICE SPARQL subquery to be executed against the resulting remote Bgee SPARQL endpoint, assuring access to the latest data. The second scenario replaces the centralized SPARQL query example with a subquery matching triple patterns from the named graph containing Bgee data and stored in the RIKEN BioResource MetaDB. We obtained the Bgee RDF data [51] on 20 July 2023 and incorporated it into the RIKEN BioResource MetaDB. To avoid longer runtimes and query timeout, we used the locally stored OMA and DisGeNET as named graphs in the BioResource MetaDB in both scenarios (Fig. 4).

**Fig. 5:**
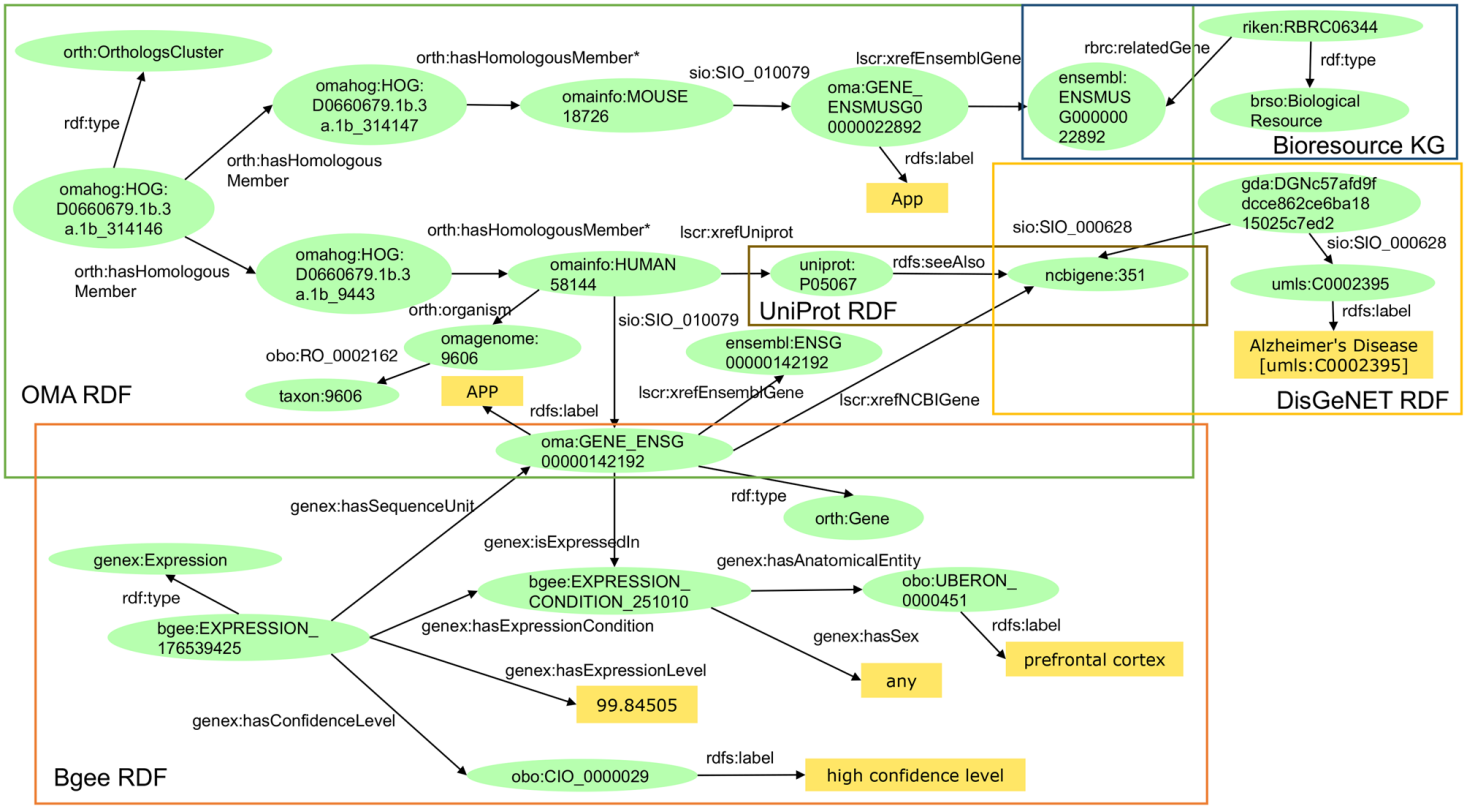
An example of the graph representation of the query result of Example 2–1 in the case of RIKEN Mouse No. RBRC06344. RBRC06344 is a knock-in mouse with a mutation inserted into the amyloid beta region of the App gene. We have added some triples (e.g., obo:UBERON_0000451 rdfs:label “prefrontal cortex”) that were not used in the query to better understand the graph

**Example 2-1**: Centralized query for Alzheimer’s disease (see Additional file 2) is based on the second scenario. Table 1 shows the query results of examples 1–1 and 2-1. The results of both were identical. The average query runtime of Example 2–1 was 307 seconds, and it was faster than that of Example 1–1 (Table 2, Fig. 5).

Namespaces

bgee: <http://bgee.org/#>

brso: < http://purl.jp/bio/10/brso/>

ensembl: < http://rdf.ebi.ac.uk/resource/ensembl/>

gda: < http://rdf.disgenet.org/resource/gda/>

genex: < http://purl.org/genex#>

lscr: <http://purl.org/lscr#>

ncbigene: < https://www.ncbi.nlm.nih.gov/gene/>

obo: < http://purl.obolibrary.org/obo/>

oma: < http://omabrowser.org/ontology/oma#>

omagenome: < https://omabrowser.org/oma/genome/>

omahog: <https://omabrowser.org/oma/hog/resolve/>

omainfo: <https://omabrowser.org/oma/info/>

orth: <http://purl.org/net/orth#>

rbrc: < http://purl.org/rbrc/resource/>

rdfs: <http://www.w3.org/2000/01/rdf-schema#>

riken: <http://metadb.riken.jp/db/rikenbrc_mouse/>

sio: < http://semanticscience.org/resource/>

taxon: < http://purl.uniprot.org/taxonomy/>

umls: < http://linkedlifedata.com/resource/umls/id/>

uniprot: <http://purl.uniprot.org/uniprot/>

We further compared federated versus centralized data access and storage approaches for other use cases. **Example 3-1** and **Example 4-1** are queries for melanoma (UMLS:C0025202) using the federated query (see Additional file 3) and centralized query (see Additional file 4) for Bgee data, respectively. These queries include the melanoma-related genes that were expressed in the skin of body (UBERON:0002097) as a query condition. The other query conditions were the same as the Examples 1–1 and 2-1.

Table 3 shows the query results of Examples 3–1 and 4-1. The findings were identical and included the demonstration that 14 genes including the HRAS gene (ENSG:00000174775) were expressed in the skin of body as melanoma-related genes, and identified 102 RIKEN bioresources were expected to be relevant to melanoma research, such as RBRC10866 [52] and RBRC01088 [53]. Table 2 shows the runtimes of Examples 3–1 and 4-1. The runtime of Example 3–1 (using a federated query) was over 600 seconds, while that of Example 4–1 (using a centralized query) was 502 seconds, which is less than the time of Example 3-1.

**Table 3:** Results of Example 3-1: Federated query for melanoma and Example 4-1: Centralized query for melanoma

Query approach	No. of retrieved mice	No. of retrieved genes	Gene labels (Ensembl Gene IDs)
Example_3-1: Federated query for melanoma	102	14	TYR (ENSG00000077498)
			PPP6C (ENSG00000119414)
			PIK3CA (ENSG00000121879)
			BRCA2 (ENSG00000139618)
			TP53 (ENSG00000141510)
			AKT1 (ENSG00000142208)
			ATM (ENSG00000149311)
			KIT (ENSG00000157404)
			TERT (ENSG00000164362)
			CTNNB1 (ENSG00000168036)
			PTEN (ENSG00000171862)
			HRAS (ENSG00000174775)
			MITF (ENSG00000187098)
			NRAS (ENSG00000213281)
Example_4-1: Centralized query for melanoma	102	14	TYR (ENSG00000077498)
			PPP6C (ENSG00000119414)
			PIK3CA (ENSG00000121879)
			BRCA2 (ENSG00000139618)
			TP53 (ENSG00000141510)
			AKT1 (ENSG00000142208)
			ATM (ENSG00000149311)
			KIT (ENSG00000157404)
			TERT (ENSG00000164362)
			CTNNB1 (ENSG00000168036)
			PTEN (ENSG00000171862)
			HRAS (ENSG00000174775)
			MITF (ENSG00000187098)
			NRAS (ENSG00000213281)

Comparing Examples 1–1 and 2-1, and 3–1 and 4-1, revealed that the query execution performance was significantly better in the centralized setup. Note that we executed the centralized queries Examples 2–1 and 4–1 for the same data as available via the remote Bgee SPARQL endpoint [54]. Thus, the significant performance differences between the federated and the centralized runtimes were not due to the Bgee data version.

Given that in our experimental setup we did not consider any engines for optimizing federated SPARQL queries [15–23], we expected that the performance of federated queries would be significantly worse than the corresponding centralized query, notably, due to network latency and poorer query optimization plan of federated queries. Large datasets such as KEGG, ChEBI, and DrugBank were benchmarked to evaluate these federated SPARQL query optimizations. However, the SPARQL queries used in the evaluation consisted of several triple patterns that were not deeply nested and had a considerably simple structure. On the other hand, the SPARQL queries (e.g., Additional file 2, and Fig. 5) used in this paper consisted of various triple patterns and were more complicated than those used in the benchmark evaluation. As a future work, we plan to carefully investigate whether the aforementioned proposed approaches would be effective in optimizing the real-world queries in this paper.

## Discussion

### Analysis and improvement of query performance

To ensure that bioresources are appropriately used as research materials in a wider range of studies, bioresource centers need to provide users with up–to-date and detailed information on the characteristics of bioresources. For this purpose, it is essential to integrate independently collected data by bioresource centers with publicly available datasets, for example, public biomedical databases. As a use case for the integration and exploitation of remote data using the Semantic Web technologies and RDF, we evaluated the performance differences between SPARQL queries by specifically examining variations in their use of subqueries and federated search techniques.

**Fig. 6:**
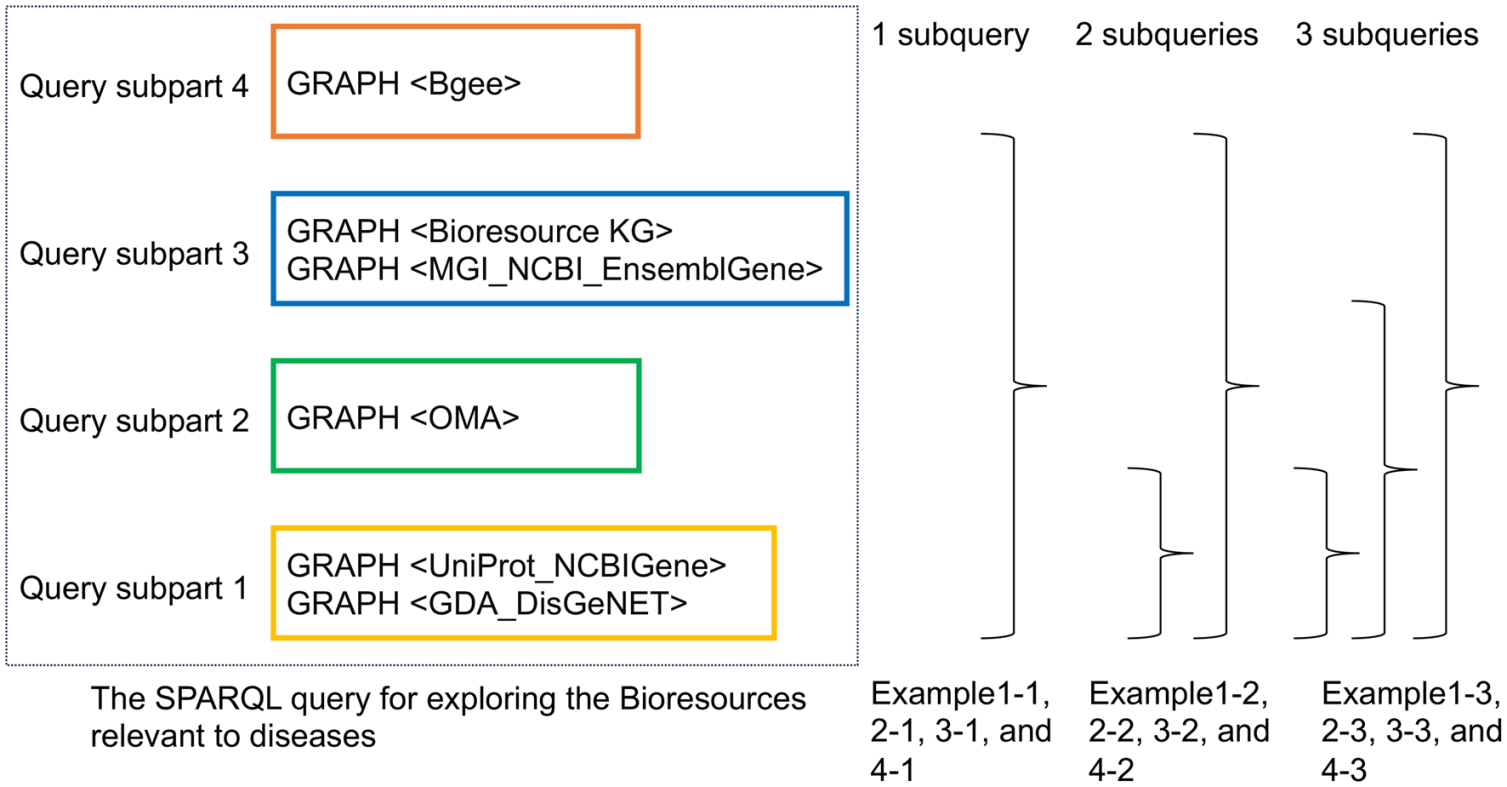
Four query subparts within the SPARQL query examples and the position of the subqueries. For the outline of query graph patterns, refer to Fig. 4

Subqueries represent a way to embed queries within other SPARQL queries, normally to achieve results which cannot otherwise be achieved, such as limiting the number of results from some sub-expression within the query [55]. The appropriate usage of subqueries is expected to improve query performance. In some cases, this is essential to avoid query timeouts and therefore to enable results to be obtained. For example, queries 1-1, 2-1, 3-1, and 4–1 contain one subquery because it would not be possible to obtain query results without the subquery due to transaction timeout (data not shown). To estimate how the usage of subqueries will affect query performance, we divided the SPARQL queries into four query subparts and investigated how the arrangement of subqueries could improve query performance (see Fig. 6). **Examples 1-2** (see Additional file 5), **2-2** (see Additional file 6), **3-2** (see Additional file 7), and **4-2** (see Additional file 8) each include two subqueries, and the remaining query conditions are the same as Example 1-1, Example 2-1, Example 3-1, and Example 4-1. **Examples 1-3** (see Additional file 9), **2-3** (see Additional file 10), **3-3** (see Additional file 11), and **4-3** (see Additional file 12) each include three subqueries, and the remaining query conditions are the same as Examples 1-1, 2-1, 3-1, and 4-1, respectively. For example, in Example 1-2, Query subpart 1 is nested inside Query subpart 2. Furthermore, Query subparts 1, 2, and 3 are nested inside Query subpart 4 (that is Bgee’s query). The nested subquery is evaluated first, and the outer query uses the results.

Table 4 shows the average runtimes of Example 1-x, 2-x, 3-x, and 4-x. Numbers highlighted in bold represent values when search results are returned within 600 seconds. For instance, in the row of Example 2-x (i.e., among Examples 2-1, 2-2, and 2-3), Example 2–1 with one subquery was the fastest, although all example queries had the same graph structures. On the other hand, we observed that the performance of the query could be significantly improved when we used the subquery in particular places, thereby providing a more effective query plan. The average runtimes for Example 2–1 (centralized for AD) and Example 4–1 (centralized for AD) were considerably lower than those for Example 1–1 (federated for AD) and Example 3–1 (federated for melanoma), respectively. The query results, such as the AD-related genes, were consistent, i.e. results of Examples 1–2 and 1–3 were the same as those of Example 1-1, and similarly for Examples 2-x and Examples 3-x. These consistent results obtained across different query formulations confirmed the appropriate use of subqueries for all Examples.

**Table 4:** The average runtime from 10 executions of the SPARQL query Examples 1-x, 2-x, 3-x, and 4-x, including one-time, twice, and three-times subqueries for the Query subparts 1 to 3, respectively

Query approach	Federated or Centralized	Target diseases	The runtime of 1 subquery (for whole of query subparts) [x = 1]	The runtime of 2 subqueries (for whole of query subparts) [x = 2]	The runtime of 3 subqueries (for whole of query subparts) [x = 3]
Example 1-x	Federated	AD	> 600 s	> 600 s	> 600 s
Example 2-x	Centralized		**307 s**	> 600 s	> 600 s
Example 3-x	Federated	melanoma	> 600 s	> 600 s	> 600 s
Example 4-x	Centralized		**502 s**	> 600 s	> 600 s

Bold values represent the best values in each row, indicating that the search results were returned within 600 s

In all Examples, we arranged Query subpart 4 (see Fig. 6) to nest other Query subparts. Next, we measured the runtimes from Query subpart 1 to 3 and that of Query subpart 4 to presume the breakdown of the runtimes. Table 5 shows the runtimes of Query subparts 1 through 3. In both the Alzheimer’s Disease (AD) and melanoma examples, we compared different query types. For AD, we have Example 5–0 without subqueries (Additional file 13), Example 5–1 with one subquery (Additional file 14), and Example 5–2 with two subqueries (Additional file 15). Similarly, for melanoma, we have Example 6–0 without subqueries (Additional file 16), Example 6–1 with one subquery (Additional file 17), and Example 6–2 with two subqueries (Additional file 18). We found that the queries with one or two subqueries (Examples 5-1, 5-2, 6-1, and 6-2) ran significantly faster than those without any subqueries (Examples 5–0 and 6-0), as shown in Table 5. These results also indicated that Query subparts 1 to 3 took 4–7 s to process.

**Table 5:** The average runtime from 10 executions of the SPARQL query Examples 5-x and 6-x, including zero, one, and two-times subqueries for the Query subparts 1 to 3, respectively

Query approach	Target diseases	The runtime of 0 subqueries (Query subpart 1, 2, and 3) [x = 0]	The runtime of 1 subquery (Query subpart 1, 2, and 3) [x = 1]	The runtime of 2 subqueries (Query subpart 1, 2, and 3) [x = 2]
Example 5-x	AD	285 s	**4 s**	**4 s**
Example 6-x	melanoma	465 s	**7 s**	**7 s**

Bold values represent the best values in each row

The numbers of retrieved bioresources and disease-related genes in Example 5-x were 56 and 15, respectively (Additional file 13, 14, and 15). The numbers of retrieved bioresources and disease-related genes of 6-x were 102 and 14, respectively (Additional file 16, 17, and 18)

Table 6 shows the runtime of Query subpart 4. The runtimes of the federated query for the prefrontal cortex [**Example 7** (Additional file 19)] and the skin of body [**Example 8** (Additional file 20)] AD-related genes were 48 and 58 s, while those of the centralized query execution for the prefrontal cortex [**Example 9** (Additional file 21)] and the skin of body [**Example 10** (Additional file 22)] were 14 and 16 s, respectively. The time differences between the federated and centralized approaches for AD and melanoma were 34 and 42 s, respectively. The retrieved bioresources and disease-related genes were the same among Examples 7 and 9, and Examples 8 and 10, respectively (Table 6).

**Table 6:** The average runtime from 10 executions of the SPARQL query Examples 7, 8, 9, and 10 without using the subqueries in Query subpart 4

Query approach	Federated or Centralized	Anatomical parts where genes were expressed	The runtime of 0 subqueries (Query subpart 4)	The difference between the Federated and the Centralized
Example 7	Federated	prefrontal cortex	48 s	34 s (between Example 7 and 9), and 42 s (between Example 8 and 10)
Example 8		skin of body	58 s	
Example 9	Centralized	prefrontal cortex	**14 s**	
Example 10		skin of body	16 s	

Bold values represent the best values in each column. We executed the federated search from the BioResource MetaDB SPARQL endpoint to the official Bgee SPARQL endpoint. We performed the centralized search from the BioResource MetaDB SPARQL endpoint to the Bgee data stored in the BioResource MetaDB

The numbers of retrieved genes in Examples 7 and 9 were the same at 42,448 (Additional file 19, and 21). The numbers of retrieved genes in Examples 8 and 10 were the same and 45,724 (Additional file 20, and 22)

Moreover, we measured the runtime of the federated approach between the BioResource MetaDB (Tsukuba in Japan) and the Bgee (Lausanne in Switzerland), and the centralized approach for Bgee data in Tsukuba and Lausanne (Table 7). We executed the centralized approaches for Bgee data stored at the RIKEN BRC (Tsukuba) and the SIB (Lausanne), from each place. The executed query includes the query conditions: the prefrontal cortex (UBERON:0000451) as the location of gene expression, a high confidence level for expression data, and the sex condition for “any sex type”. As a result, the runtime of the federated approach (Tsukuba to Lausanne) was 48 s, including data transfer time and the Bgee triple store query evaluation time. The centralized approach runtime in Lausanne (Lausanne to Lausanne) was 11 s, and that in Tsukuba (Tsukuba to Tsukuba) was 14 s. From these results, we estimated the data transfer time between Tsukuba and Lausanne was 37 s (the column of [A–B] in Table 7), and the difference between the query evaluation time of the BioResource MetaDB in Tsukuba and Bgee in Lausanne was 3 seconds (the column of [C–B] in Table 7).

**Table 7:** Comparison of the runtimes of the federated approach from the BioResource MetaDB (Tsukuba) to the Bgee (Lausanne), and the centralized approach at Tsukuba and Lausanne

Query approach	Federated or Centralized	No. of retrieved genes	Mean of runtime	[A–B]	[C–B]
Federated (Tsukuba to Lausanne): Example 7	Federated	42,448	48 s [A]	37 s	
Centralized (Lausanne to Lausanne)*	Centralized	42,448	11 s [B]		3 s
Centralized (Tsukuba to Tsukuba): Example 9		42,448	14 s [C]		

We conducted the performance by executing the SPARQL query for Bgee data (Examples 7 and 9). The executed query includes the query conditions: the prefrontal cortex (UBERON:0000451) as location of gene expression, a high confidence level for expression data, the sex condition for “any sex type”

*: After we removed a row including the SERVICE keyword from Example 7 and executed it

A–B: Data transfer time between Tsukuba (RIKEN BRC) and Lausanne (Bgee)

C–B: The difference in the retrieval time of the BioResource MetaDB and Bgee

From the results of Tables 5, 6, and 7, we concluded that one of the reasons for the query performance degradation in the federated approach and the improvement was as follows, (1) the difference in the total runtime of the federated and centralized approach (e.g., 34 seconds between Examples 7 and 9 in Table 6) mainly depended on the data transfer time between Tsukuba and Lausanne and the query evaluation time of Query subpart 4 (Bgee data) since the runtime of Query subpart 1 (see Fig. 6) through 3 took 4–7 s by using the subqueries (Examples 5-1, 5-2, 6-1, and 6–2 in Table 5). (2) We estimated the data transfer time between Tsukuba and Lausanne took 37 seconds (the column of [A–B] in Table 7). At this time, the number of data transferred from Tsukuba to Lausanne was 42,448 genes (Table 7). Table 8 shows the execution time when the LIMIT and OFFSET modifiers in SPARQL were used to limit the number of search results to 100 rows (genes) in Examples 7 and 9, as well as in the centralized approach in Laurence. We estimated the total time, including data transfer between Tsukuba (RIKEN BRC) and Lausanne (Bgee), and the time to display search results to be 2 s (the column of [A–B] in Table 8). The difference between the query evaluation time of the BioResource MetaDB in Tsukuba and Bgee in Lausanne was 3 s (the column of [C–B] in Table 8). We found that the data transfer time was reduced since we refined the quantity of transferred data from Tsukuba to Lausanne by using subqueries and SPARQL’s LIMIT and OFFSET modifiers (see Additional file 19, and Additional file 21). (3) On the other hand, the query evaluation times of Bgee data (Query subpart 4) in the BioResource MetaDB (Tsukuba) and the Bgee database (Lausanne) took 11 ([B] in Table 7) and 14 seconds ([C] in Table 7), respectively, and the difference between them was 3 s (the column of [C–B] in Table 7).

**Table 8:** Comparison of the runtimes of the federated approach from the BioResource MetaDB (Tsukuba) to the Bgee (Lausanne), and the centralized approach at Tsukuba and Lausanne

Query approach	Federated or Centralized	No. of retrieved genes	Mean of runtime	[A–B]	[C-B]
Federated (Tsukuba to Lausanne): Example 7	Federated	100	3 s [A]	2 s	
Centralized (Lausanne to Lausanne)*	Centralized	100	1 s [B]		3 s
Centralized (Tsukuba to Tsukuba): Example 9		100	4 s [C]		

We conducted the performance by executing the SPARQL query for Bgee data (Examples 7 and 9). The executed query includes the query conditions: the prefrontal cortex (UBERON:0000451) as location of gene expression, a high confidence level for expression data, the sex condition for “any sex type”. Furthermore, we used the LIMIT and OFFSET modifiers in SPARQL to limit the number of search results to 100 rows (genes)

*: After we removed a row including the SERVICE keyword from Example 7 and executed it

A–B: Data transfer time between Tsukuba (RIKEN BRC) and Lausanne (Bgee)

C-B: The difference in the retrieval time of the BioResource MetaDB and Bgee

As a result, the reasons for the differences depend on the server’s specification (e.g., the memory capacity) and the database type (e.g., Virtuoso), the versions, the settings, and scalability issues. Therefore, we could improve the degradation of the query performance of the federated approach from the BioResource MetaDB to the SIB by enhancing the server specifications and by optimizing the triple store. First, we questioned whether the longer runtimes in the federated approach could be caused by network latency (Section “Comparison Between Federated and Centralized Query Performance”), and asked whether its extent could be mitigated by reducing the quantity of data transfer during the execution of subqueries. Indeed, by optimizing the evaluation of triple store queries in Bgee’s triple store as an additional query performance test, the execution times of Examples 1–1 and 3–1 for the federated approach were improved to the same level as that of Examples 2–1 and 4–1 for the centralized approach. (see README.md in this project [56]).

In addition, using the federated search exhibited several important advantages. For institutions such as the RIKEN BRC, which combines its own RDF data with external datasets, using the federated approach should leverage the latest, most up–to-date information from each external dataset and thereby reduce operational costs that would be required to maintain a local copy in-sync when the external sources are updated. The federated approach is therefore particularly beneficial for institutions that use multiple third-party datasets. The federated approach is an essential technology for exploring bioresources relevant to biomedical research, which requires the combination of several external datasets.

### Execution of a transitive search using external data

**Fig. 7:**
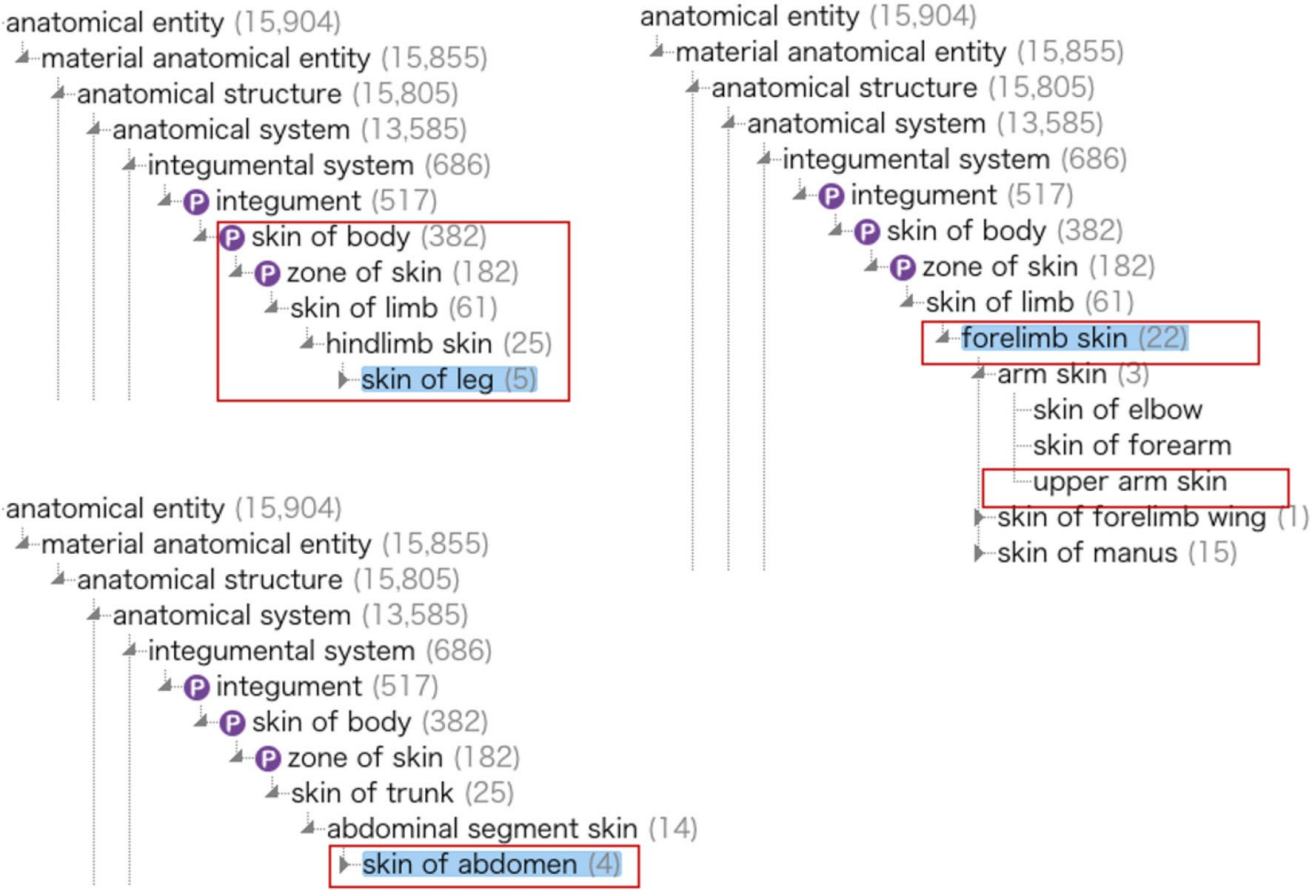
A part of the ontological tree of the Uberon ontology. Red rectangles indicate anatomical sites where melanoma-related genes were expressed. The “P” mark represents the “part of” relation. This ontological tree was made from a diagram of the Ontology Lookup Service (OLS) at https://www.ebi.ac.uk/ols4

This study used Uberon ontology terms, such as prefrontal cortex (UBERON:0000451) or skin of body (UBERON:0002097), as anatomical parts where genes are expressed. However, we realized we could not comprehensively acquire expressed genes at specific anatomical locations using the example queries shown so far. In Examples 3–1 and 4-1, we specified the “skin of body” as the target anatomical parts and observed genes expressed at those anatomical parts. However, in these cases, we cannot find expressed genes on the “zone of skin” (UBERON:0000014) that is a part of “skin of body” or on the “skin of limb” (UBERON:0001419) that is subClassOf “zone of skin” (Fig. 7). When the users specify “skin of body” as target anatomical parts, they would often expect to acquire expression information from both the “skin of body” and the subclass concepts that are subClassOf or part of “skin of body.”

Balhoff et al. [33] cited “index_finger is_a finger” and “finger part_of_hand” as examples and they mentioned that a user would expect that when querying for parts of the hand they would receive not only ‘finger’ but any concepts stated to be parts thereof (e.g., fingernails) or subclasses of ‘finger,’ and SPARQL property paths cannot be easily employed to retrieve nodes linked by a chain of properties over such OWL expressions. Furthermore, OBO library ontologies include a wealth of inter-ontology semantic links, which require OWL reasoning to be fully utilized. One way to accomplish this would be to import all the needed ontologies into the Protégé tool [57, 58] or an RDF store with an inference engine such as Stardog [59], and run an OWL reasoner, while it will need to be aware of the OWL-RDF serialization in order to match these complex triple patterns. Subsequently, they developed the Ubergraph, which currently includes 39 OBO ontologies including the Uberon with precomputed relations, to solve this issue by performing SPARQL queries that make use of the semantics of the included ontologies [33].

On the other hand, we strove to solve the problem of mixed subClassOf and partOf relationships between anatomical terms in Uberon, where the depth of the hierarchy is unknown, by reusing existing public resources and using SPARQL. We acquired the latest uberon_kgx_tsv_edge.tsv [60] that was published from the KG-OBO project and converted the downloaded tsv format file to two turtle (ttl) format files by a Python script (see Additional file 23). The uberon_kgx_tsv_edge.tsv was a KGX TSV format file by being transformed from uberon.owl [61] using the Koza tool [24]. Our converted two ttl format files included subject_broader_object_from_BFO_0000050.ttl (see Additional file 24) and subject_broader_object_from_subClassOf.ttl (see Additional file 25). The former file was converted from part of the relation between subject and object terms to the “broader” predicate [62], the latter file was converted from subClassOf relation to the “broader” predicate. The broader relation is a predicate directly connecting among uberon terms instead of partOf and subClassOf relations. We stored these two ttl format files as a named GRAPH: <http://metadb.riken.jp/db/uberonRDF_broader_fromKGX> into the BioResource MetaDB. We term these two ttl format data the uberonRDF-KGX.

**Fig. 8:**
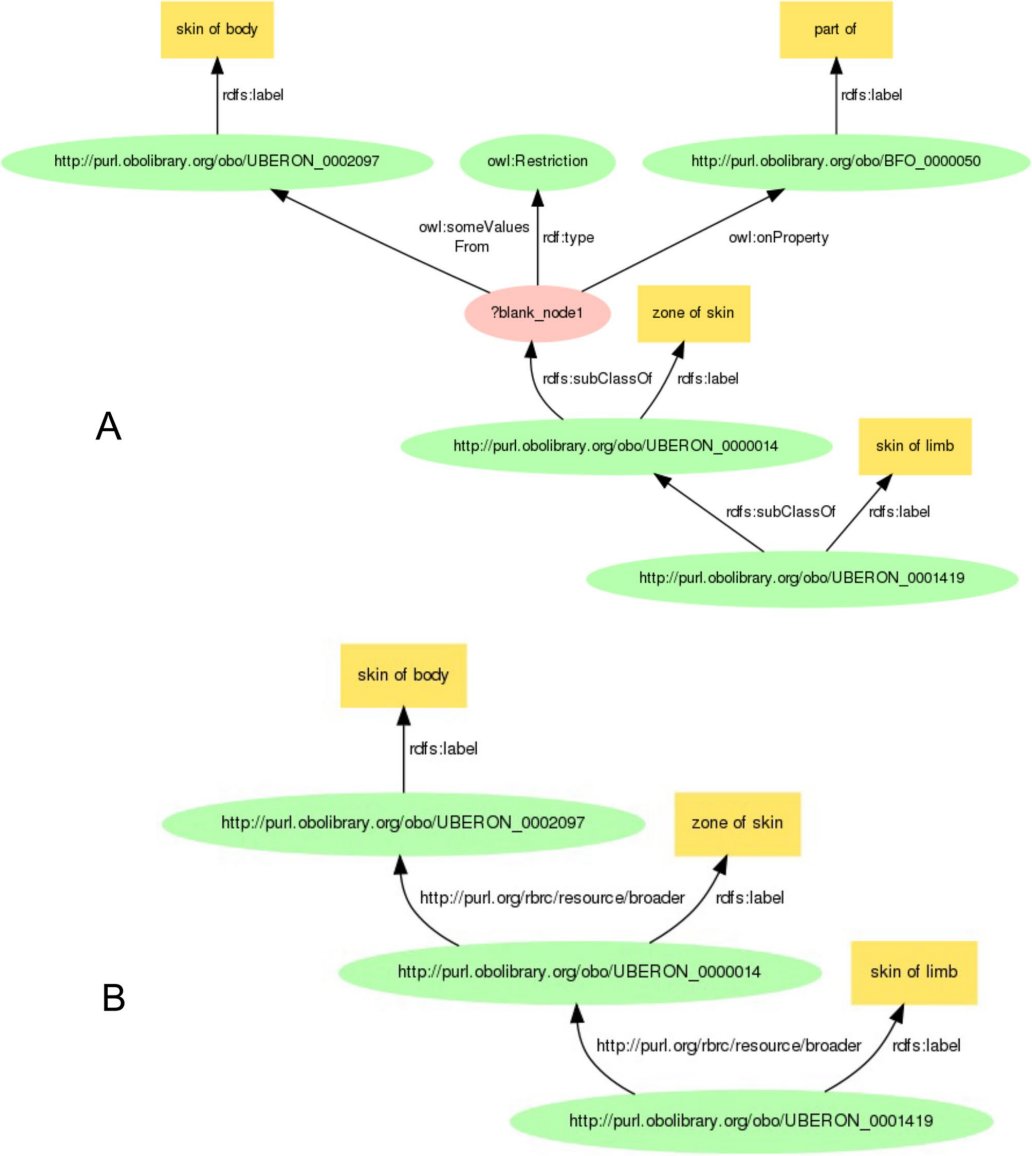
A path between the “skin of limb” (UBERON:0001419) and the “skin of body” (UBERON:0002097) in the uberon.owl (**A**) and that in the named GRAPH < http://metadb.riken.jp/db/uberonRDF_broader_fromKGX> within the RIKEN BioResource MetaDB (**B**). These diagrams were created using https://www.kanzaki.com/works/2009/pub/graph-draw

Figure 8 demonstrates a path between the “skin of limb” (UBERON:0001419) and the “skin of body” (UBERON:0002097) in the uberon.owl (diagram A) and the named GRAPH <http://metadb.riken.jp/db/uberonRDF_broader_fromKGX> (diagram B) within the RIKEN BioResource MetaDB. In the uberon.owl (diagram A), the “skin of body” connects to the “skin of limb” through the rdfs:subClassOf and owl:someValueFrom, while in the diagram B, the “skin of body” connects to the “skin of limb” through two broader predicates. Since it is difficult to execute a transitive search among Uberon terms by using the SPARQL query for uberon.owl (diagram A), we successfully executed a transitive search by using the Property Paths function of SPARQL query for the named GRAPH <http://metadb.riken.jp/db/uberonRDF_broader_fromKGX> (diagram B), whereby data was converted from part of and subClassOf relations to the broader predicate.

**Example 11-1:** Centralized query for melanoma using the uberonRDF-KGX (see Additional file 26) is a SPARQL query where we added the named GRAPH: <http://metadb.riken.jp/db/uberonRDF_broader_fromKGX> to the Example 4–1 so as to execute a transitive search for the Uberon terms by using the Property Paths function.

**Example 11-2**: Federated query for melanoma using the Ubergraph data instead of the uberonRDF-KGX is a SPARQL query (see Additional file 27). This query includes a service keyword to execute a transitive search for Uberon RDF data in the Ubergraph through the federated approach to the Ubergraph SPARQL endpoint [63]. In advance, we performed a preliminary test for Examples 11–1 and 11-2, identifying the same results.

Table 9 shows the average runtimes of Examples 11–1 and 11-2. The runtime of Example 11–1 was 627 s, on the other hand, we did not obtain the result of Example 11–2 due to a transaction timeout (over 3600 s). Table 10 shows the query result of Example 11-1. We found 14 genes including the HRAS gene (ENSG:00000174775) and PTEN gene (ENSG:00000171862), which were expressed in the “skin of body” or 12 anatomical locations that comprise the partOf or subClassOf the skin of body (Table 10). HRAS and PTEN genes are highly relevant for melanoma research, as shown in [64, 65]. The anatomical locations on which the 14 genes were expressed include 12 locations, such as the skin of limb and forelimb skin (UBERON:0003531) in addition to the skin of body (Table 10, Fig. 7). Furthermore, we explored 102 RIKEN bioresources expected to be suitable for melanoma research (Table 10). Specifying ‘skin of body’ as a query condition (Example 11-1), we identified melanoma-associated genes, the gene expression levels, each gene expression site (e.g., ‘skin of limb’, a narrower term of ‘skin of body’), and bioresources predicted to be suitable for melanoma research (Additional file 26, Figs. 7 and 8). We concluded that this is because Example 11–1 could execute a transitive search for the Uberon data using the SPARQL query’s Property Paths function.

**Table 9:** The average runtime from 10 executions of the SPARQL query Examples 11–1 and 11-2

Query approach	Target diseases	Which Uberon data was used	Mean of runtime
Example 11-1	melanoma	The Uberon data converted from KGX stored in the BioResource MetaDB (Centralized approach)	627 s
Example 11-2		The Ubergraph data through the SPARQL endpoint (federated approach)	Transaction timed out (over 3600 s)

**Table 10:** Results of Example 11-1: Centralized query for melanoma using the broader predicate to perform the property path function

Query approach	No. of retrieved mice	No. of retrieved genes	Gene labels (Ensembl Gene IDs)	No. of anatomical entities	Anatomical entity labels (Uberon IDs)
Example_11-1: Centralized query for melanoma using the broader predicate	102	14	TYR (ENSG00000077498) PPP6C (ENSG00000119414) PIK3CA (ENSG00000121879) BRCA2 (ENSG00000139618) TP53 (ENSG00000141510) AKT1 (ENSG00000142208) ATM (ENSG00000149311) KIT (ENSG00000157404) TERT (ENSG00000164362) CTNNB1 (ENSG00000168036) PTEN (ENSG00000171862) HRAS (ENSG00000174775) MITF (ENSG00000187098) NRAS (ENSG00000213281)	12	zone of skin (UBERON_0000014) skin epidermis (UBERON_0001003) skin of abdomen (UBERON_0001416) skin of limb (UBERON_0001419) skin of leg (UBERON_0001511) skin of hip (UBERON_0001554) hair follicle (UBERON_0002073) skin of body (UBERON_0002097) forelimb skin (UBERON_0003531) hindlimb skin (UBERON_0003532) upper leg skin (UBERON_0004262) upper arm skin (UBERON_0004263)

## Future work

The bioresource KG integrated with OMA, DisGeNET, Bgee enable bioresource users, such as medical researchers and experimental researchers, to efficiently obtain accurate and comprehensive information on the disease-related human genes, gene expression levels at any anatomical parts, and the related experimental mice of their interested disease at once. The distribution of high-quality bioresources, which serve as research platforms, contributes to the development of biomedical research. In this paper, we only shared information on disease model mice, but the KG also included gene materials (e.g., disease-related cDNA clones) and cell materials (e.g., patient-derived iPS cells) [9]. As a result, bioresource users can simultaneously acquire these different types of bioresources, namely mice, cells, and DNA materials related to any diseases, thanks to the integrated KG. Furthermore, by combining other bioresource or model organism data, such as a rat, Xenopus, and zebrafish from external institutes, we could find novel disease model organisms through GeneIDs, disease ontology, and phenotype terms.

In the demonstration of Section “Exploring Bioresources Relevant to Human Diseases”, we only used the DisGeNET as a GDA dataset. However, in the preliminary trials we performed, we successfully demonstrated the use of other datasets, such as MedGen, and MGI, instead of the DisGeNET (see this project webpage [66]). Therefore, we can select one of these GDA datasets or combine several. In the latter case, we can use common (intersection of) GDA data among DisGeNET, MedGen, and MGI datasets. In addition, the integration of the Bgee dataset allows us to handle information on gene expression levels at specific anatomical locations. The Bgee dataset includes the development stage (e.g., late adult stage), sex, strain, and data source (e.g., RNA Seq) in addition to the anatomical location. The use of Bgee gene expression data is expected to lead to the exploration of more specific disease-related genes and bioresources.

In this article, we introduced a method to explore bioresources used for specific disease research using SPARQL queries. However, not all users of bioresources can perform information retrieval using SPARQL. Furthermore, the SPARQL query’s runtime sometimes takes several hundreds of seconds depending on the query conditions (Tables 2 and 4), and we observed that it needs to be shorter to provide efficient retrieval results for users. Therefore, we have developed a keyword search engine and interface for bioresource users and have accomplished a few seconds of runtime. The Search for bioresources tab [2, 67] leverages the technology of SPARQList [68], which provides a REST API server for a SPARQL query against bioresource association data collected by crawling the KG (Fig. 1) [69] and is a bioresource search service that enables keyword search using disease name, gene name, resource name, and species name. We plan to expand the keyword search function in the Search for bioresources tab to enable searching by the Uberon Ontology term. Moreover, we are also developing an interface that allows users to select ontology terms from the ontology tree structure so as to search for the related bioresources.

## Electronic supplementary material

Below is the link to the electronic supplementary material.

Supplementary Material 1

Supplementary Material 2

Supplementary Material 3

Supplementary Material 4

Supplementary Material 5

Supplementary Material 6

Supplementary Material 7

Supplementary Material 8

Supplementary Material 9

Supplementary Material 10

Supplementary Material 11

Supplementary Material 12

Supplementary Material 13

Supplementary Material 14

Supplementary Material 15

Supplementary Material 16

Supplementary Material 17

Supplementary Material 18

Supplementary Material 19

Supplementary Material 20

Supplementary Material 21

Supplementary Material 22

Supplementary Material 23

Supplementary Material 24

Supplementary Material 25

Supplementary Material 26

Supplementary Material 27

## Data Availability

All materials and data of this paper, including SPARQL query examples and results, are published as Additional Files. Other materials and data are available from the corresponding author upon request.

## Abbreviations

AD: Alzheimer’s disease
BRC: BioResource Research Center
GDA: Gene Disease Associations
KG: Knowledge Graphs
MGI: Mouse Genome Informatics
OMA: Orthologous MAtrix
RDF: Resource Description Framework
