# Supplementary material for: Federated SPARQL query performance evaluation for exploring disease model mouse: combining gene expression, orthology, and disease knowledge graphs
Source: BMC Med Inform Decis Mak. 2025 May 16;25(Suppl 1):189. doi: 10.1186/s12911-025-03013-8 (PMC12082848; doi:10.1186/s12911-025-03013-8)
Supplement: Supplementary file 4 — Supplementary Material 4 [file 12911_2025_3013_MOESM4_ESM.docx]

**Additional file 4**

<https://github.com/kushidat/broaderPredicate_uberon?tab=readme-ov-file#additional-file-4-sparql-query-example-4-1>

Query Example 4-1 (A centralized SPARQL query for melanoma with one subquery) and the query result.
