# Supplementary material for: Federated SPARQL query performance evaluation for exploring disease model mouse: combining gene expression, orthology, and disease knowledge graphs
Source: BMC Med Inform Decis Mak. 2025 May 16;25(Suppl 1):189. doi: 10.1186/s12911-025-03013-8 (PMC12082848; doi:10.1186/s12911-025-03013-8)
Supplement: Supplementary file 19 — Supplementary Material 19 [file 12911_2025_3013_MOESM19_ESM.docx]

**Additional file 19**

<https://github.com/kushidat/broaderPredicate_uberon?tab=readme-ov-file#additional-file-19-sparql-query-example-7>

Query Example 7 (A federated SPARQL query of Query subpart 4 (Bgee) for the expressed genes in the "prefrontal cortex") and the query result
