# Supplementary material for: Federated SPARQL query performance evaluation for exploring disease model mouse: combining gene expression, orthology, and disease knowledge graphs
Source: BMC Med Inform Decis Mak. 2025 May 16;25(Suppl 1):189. doi: 10.1186/s12911-025-03013-8 (PMC12082848; doi:10.1186/s12911-025-03013-8)
Supplement: Supplementary file 23 — Supplementary Material 23 [file 12911_2025_3013_MOESM23_ESM.docx]

**Additional file 23**

https://github.com/kushidat/broaderPredicate_uberon?tab=readme-ov-file#additional-file-23-python-script

This is a Python script for converting the latest uberon_kgx_tsv_edge.tsv from the kg-uberon webpage in the KG-OBO project (<https://kg-hub.berkeleybop.io/kg-obo/uberon/>) to two ttl format files, including subject_broader_object_from_BFO_0000050.ttl (Additional file 24) and subject_broader_object_from_subClassOf.ttl (Additional file 25).
