# Supplementary material for: Federated SPARQL query performance evaluation for exploring disease model mouse: combining gene expression, orthology, and disease knowledge graphs
Source: BMC Med Inform Decis Mak. 2025 May 16;25(Suppl 1):189. doi: 10.1186/s12911-025-03013-8 (PMC12082848; doi:10.1186/s12911-025-03013-8)
Supplement: Supplementary file 25 — Supplementary Material 25 [file 12911_2025_3013_MOESM25_ESM.docx]

**Additional file 25**

https://github.com/kushidat/broaderPredicate_uberon?tab=readme-ov-file#additional-file-25-rdf-data

This is a turtle file converted from the latest uberon_kgx_tsv_edge.tsv in the kg-uberon webpage in the KG-OBO project (https://kg-hub.berkeleybop.io/kg-obo/uberon/). This file includes the broader predicate’s relationships among terms created from the rdfs:subClassOf relations in the uberon_kgx_tsv_edge.tsv in the kg-uberon.
